# MR1-dependence of unmetabolized folic acid side-effects

**DOI:** 10.3389/fimmu.2022.946713

**Published:** 2022-08-09

**Authors:** Jeffry S. Tang, Alissa Cait, Reuben M. White, Homayon J. Arabshahi, David O’Sullivan, Olivier Gasser

**Affiliations:** ^1^ Malaghan Institute of Medical Research, Wellington, New Zealand; ^2^ High-Value Nutrition National Science Challenge, Auckland, New Zealand; ^3^ School of Chemical Sciences, University of Auckland, Auckland, New Zealand

**Keywords:** MR1, MAIT cell, folate, folic acid (B9), unmetabolized folic acid

## Abstract

The fortification of flour with folic acid for the prevention of neural tube defects (NTD) is currently mandated in over eighty countries worldwide, hence compelling its consumption by the greater part of the world’s population. Notwithstanding its beneficial impact on rates of NTD, pervasive folic acid supplementation has invariably led to additive daily intakes reaching well beyond their original target, resulting in the circulation of unmetabolized folic acid. Associated idiopathic side-effects ranging from allergies to cancer have been suggested, albeit inconclusively. Herein, we hypothesize that their inconsistent detection and elusive etiology are linked to the *in vivo* generation of the immunosuppressive folic acid metabolite 6-formylpterin, which interferes with the still emerging and varied functions of Major Histocompatibility Complex-related molecule 1 (MR1)-restricted T cells. Accordingly, we predict that fortification-related adverse health outcomes can be eliminated by substituting folic acid with the bioequivalent folate vitamer 5-methyltetrahydrofolate, which does not break down into 6-formylpterin.

## Introduction

Folate, a group denomination for a range of chemically distinct compounds collectively known as vitamin B9, is critical for cellular function and development. Folate acts as a co-factor in one-carbon metabolism and exists in methylated and unmethylated forms (1). Folate is required during pregnancy for correct neural tube development, and supplementation or dietary fortification with folate is well documented to reduce the risk of neural tube defects (NTD) (2). Importantly, folate fortification is almost exclusively implemented through folic acid (FA), an unmethylated and oxidized, synthetic form of folate (3). While low doses of FA are readily reduced and methylated by the intestinal epithelium or the liver, doses >200 μg – for reference a single serve of breakfast cereals can contain up to 400 μg – lead to the appearance of unmetabolized FA (UMFA) in blood and cord blood (4, 5). As folate primarily circulates as 5-methyltetrahydrofolate (5-MTHF) in human blood (6), the occurrence of UMFA is unnatural and challenges the safety of excess folic acid intake (7–11). High folate/UMFA has repeatedly been associated with adverse cancer, metabolic, cognitive and hypersensitivity-related outcomes (11–21), but an equally large body of evidence calls these observations into question (22–28). A recent consensus among experts indicates that the enigmatic side-effects of UMFA, should they exist, likely involve noncanonical pathways unrelated to one-carbon metabolism, highlighting the pressing need to investigate alternative mechanisms of action (8).

Independently, the FA breakdown product and photometabolite 6-formylpterin (6-FP), due to its ability to bind and stabilize Major Histocompatibility Complex-related molecule 1 (MR1), contributed to the emergence of the rapidly growing field of MR1 immunobiology (29, 30). The functional characterization of the largest MR1-restricted T (MR1-T) cell subset, mucosal-associated invariant T (MAIT) cells, has since generated tremendous scientific interest (31); the biological relevance of 6-FP’s (or 6-FP synthetic analogues’) inhibitory roles on MAIT cells and antitumoral MR1-T cells comparably little (32–36). Proceeding on our recent observations demonstrating the immunosuppressive potency of folic acid (photo)degradation *in vivo* (37–39), we propose that MR1 immunobiology may hold the key to solve the problems surrounding FA fortification.

## MR1-restricted T cells

**Figure 1:**
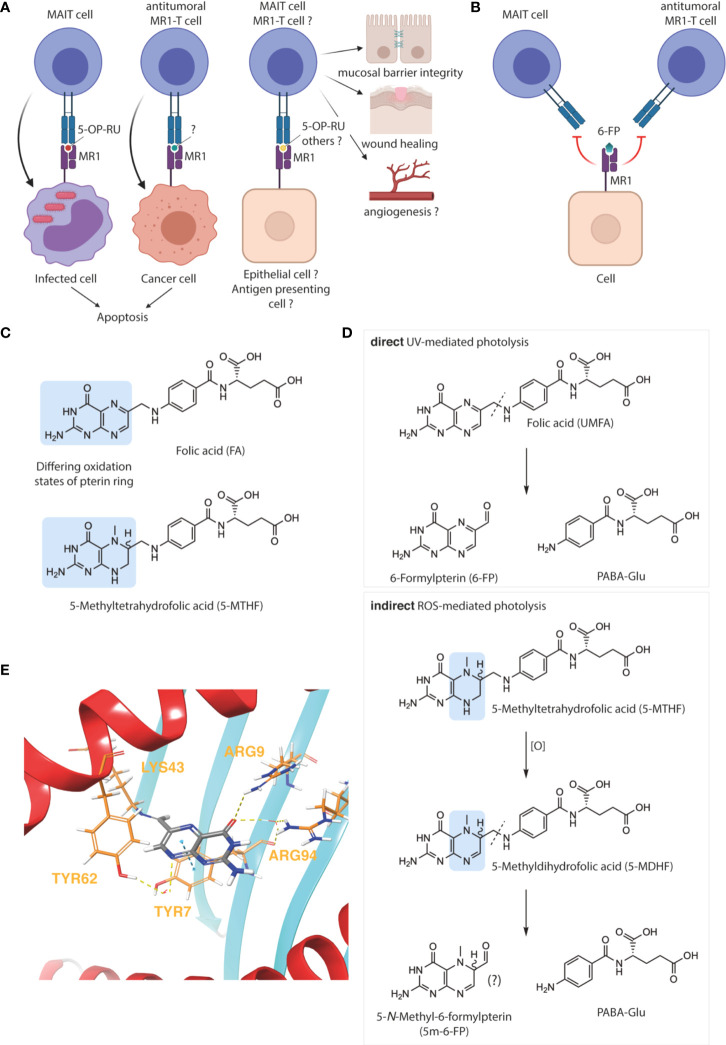
Oxidative degradation of folate and its relevance for MR1 immunobiology. MR1–restricted T cells have varied biological functions **(A)**, which are blocked by 6–formylpterin (6–FP) **(B)**, image created with BioRender. The folate vitamers folic acid (FA) and 5–methyltetrahydrofolate (5–MTHF) differ in their pterin ring and oxidation states, highlighted in blue **(C)**. FA undergoes direct photodegradation to 6–FP and *p*–aminobenzoyl glutamic acid (PABA–Glu) upon UVA exposure (**D**, upper panel). In contrast, the photodegradation of 5–MTHF is indirect and likely mediated by reactive oxygen species (ROS). The pterin photo–metabolite of 5–MTHF has not yet been formally characterized but could hypothetically be 5–*N*–Methyl–6–formylpterin (5m–6–FP) (**D**, lower panel). Binding mode of 6–FP to MR1 (PDB ID: 4L4T) was visualized using the Maestro software suite **(E)**. The protein was prepared using the Schrodinger protein preparation wizard and docked using CovDock. Key MR1 amino acid residues interacting with 6–FP (gray), either covalently (LYS43) or non–covalently (ARG9, ARG94, TYR7 and TYR62), are marked in yellow and non–covalent interactions represented by dashed lines. H–bond interactions between various contact points of 6–FP with ARG9, ARG94, or TYR62 (*via* a water molecule) are represented by dashed yellow lines. The π–stacking interaction between the pterin ring of 6–FP with the aromatic ring of TYR7 is represented by a dashed blue line **(E)**.

The main subset of MR1-T cells, MAIT cells, are largely characterized as antibacterial (31). This function is afforded by the specificity of MAIT cells’ semi-invariant T cell receptor (TCR) for MR1-bound 5-(2-oxopropylideneamino)6-D-ribitylaminouracil (5-OP-RU) (**Figure 1A**), which is formed upon condensation of the microbial riboflavin intermediate 5-amino-6-D-ribitylaminouracil (5-A-RU) with endogenous methylglyoxal (40). Recent observations indicate that MR1-restricted recognition of 5-OP-RU can also be mediated by non-invariant TCRs, thereby broadening the definition of MAIT cells and the family of MR1-restricted T cells more generally (31, 41, 42). Additional roles in tissue homeostasis and mucosal barrier function have recently become evident, highlighting the role of MAIT cells as mediators of host-microbe interactions in the context of both microbial infection and commensalism. Other suggested functions include tissue repair and wound healing, angiogenesis, and metabolic homeostasis (**Figure 1A**) (31, 43–45).

Due to MAIT cells’ microbial specificity, their sterile, homeostatic range of activities may be rapidly revoked upon bacterial infection. The hypothesis presented herein emerged while investigating the function of MAIT cells as pathogenic drivers of atopic dermatitis (AD), through the pro-inflammatory sensing of bacterial translocation across the skin barrier (39). In that setting, we observed ultraviolet light (photo-)therapy, second-line treatment for human AD, to be MR1- and FA-dependent and mediate its therapeutic effect through the 6-FP-mediated blockade of MR1, reducing its ability to present of 5-OP-RU to MAIT cells. Importantly, due to the limiting amounts of circulating FA available for photolysis, the therapeutic benefit of phototherapy was significantly enhanced by topical application of FA prior to phototherapy (39). Topical FA also reduced disease burden without subsequent phototherapy, likely due to storage-related degradation into 6-FP. In contrast, 5-MTHF did not show any activity in either setting.

While 6-FP-mediated MAIT cell inhibition may thus be contextually beneficial, and may provide protection in other settings where MAIT cells have been described to exert pathogenic activity (31), our hypothesis represents a logical extrapolation of our observations to immune homeostasis under steady state, and its potential undermining by supraphysiological levels of what could be defined as the proform of a MR1-specific immunosuppressant (i.e. UMFA). In that context, the impairment of MAIT cell-dependent mucosal integrity becomes a conceivable etiology for the association between high folate – or high UMFA to 5-MTHF ratio – and allergic disease (**Figure 1B**) (15, 19). Other side-effects of UMFA/6-FP may in turn be mediated through the inhibition of non-MAIT MR1-T cell subsets. Preeminently, the potential pro-tumorigenic effects of UMFA/6-FP may occur through the inhibition of cytotoxic, cancer-specific MR1-T cells (**Figure 1B**) (34, 46). More speculatively, 6-FP may have an immune-activatory function through the engagement of yet-to-characterized non-classical MAIT cells or atypical MR1-T cells (31, 42).

As the field of MR1 immunobiology continues to mature, so will the understanding of the physiological relevance of 6-FP as related to UMFA. Of particular concern is the enrichment, yet unknown function, of MAIT cells in the intervillous space of the placenta (47).

## Folic acid *vs* 5-methyltetrahydrofolate

FA and 5-MTHF represent the fully oxidized unmethylated and fully reduced methylated forms of folate, respectively (**Figure 1C**). Due to this fundamental difference and the physicochemical implications briefly discussed below, the substitution of FA with 5-MTHF – which increases blood folate indices to a similar extent and is an accepted alternative to prevent NTD – is apt to prevent the generation of 6-FP and/or interference with MR1-T cell function (48, 49). An important and comforting corollary to our hypothesis is therefore that the adverse health outcomes associated with folate fortification are folic acid-specific and not due to the over-consumption of folate *per se*. Thus, substitution of FA with 5-MTHF in fortified foods would be predicted to resolve idiopathic negative health effects associated with folate fortification.

As accentuated by therapeutic and evolutionary considerations (50–52), the oxidative degradation of folate predominantly occurs in the skin. This is attributable to the propensity of folate and other pterin-based molecules to absorb light in the ultraviolet (UV) spectrum. However, compared to 5-MTHF, FA absorbs significantly more ultraviolet A (UVA), the major type of UV radiation penetrating the earth’s atmosphere and human skin, making FA more susceptible to photo-oxidation when circulating through the dermal capillary bed (**Figure 1D**) (53, 54). Significant FA photodegradation, resulting in a net decrease of total blood folate, can indeed be observed after a single 2 hour sunlight exposure in healthy individuals with circulating UMFA, whilst 5-MTHF is comparably photostable (55).

**Figure 2:**
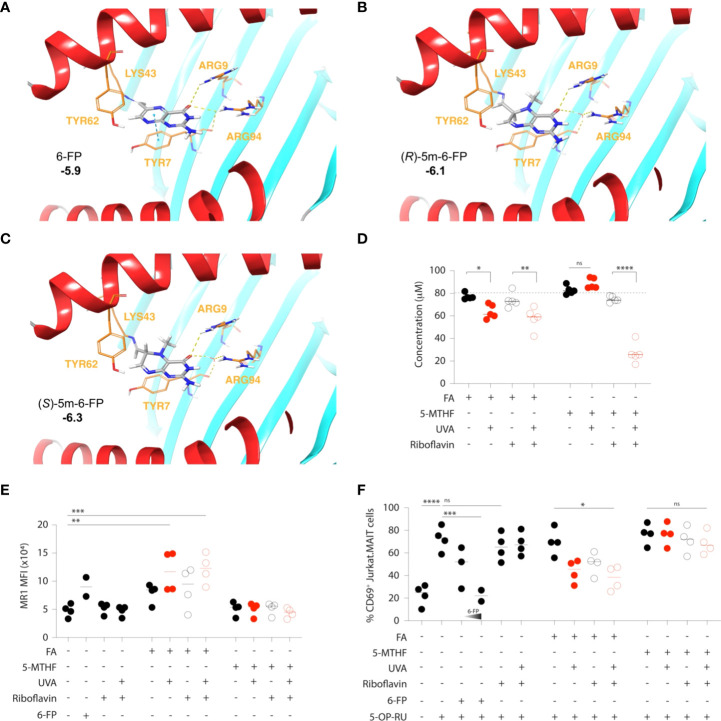
Inhibition of MAIT cell activation by folic acid, but not 5–methylfolate, photometabolites. **(A–C)** Docked conformation of 6–FP **(A)**, (*R*)–5m–6–FP **(B)** and (*S*)–5m–6–FP **(C)** to the MR1 binding site. Cdock affinity scores are highlighted in bold. The carbonyl of 6–FP interacts with the positively charged residues ARG9 *via* hydrogen bonding and ARG94 *via* water mediated hydrogen bonding. A π–stacking interaction with TYR7 is also observed. The (*R*) and (*S*) 5–m–6–FP enantiomers show similar positioning within the pocket but have no π–stacking interaction with TYR7. Ligand carbons are marked in grey while protein carbons are marked in orange. Hydrogen bonding and hydrophobic interactions are depicted as yellow and blue dashed lines, respectively. **(D–F)** Folic acid (FA) and 5–methyltetrahydrofolate (5–MTHF) solutions (80 μM in PBS) were exposed to UVA radiation in the presence or absence of equimolar amounts (80 μM) of the natural photosensitizer riboflavin. The extent of their respective photodegradation was determined by UV–Vis spectrophotometry **(D)**. The release of inhibitory MR1 ligands was assessed by the staining intensity (as mean fluorescence intensity, MFI) of cell surface MR1 on antigen presenting (C1R.MR1) cells **(E)** and the associated inhibition of 5–OP–RU–mediated activation of a MAIT cell line (Jurkat.MAIT), as determined by the expression of the activation marker CD69 **(F)**. 6–FP (5 μM and 10μM) was used as a control. Each symbol represents the mean value of an independent experiment (2–4), each run in triplicates. Statistics were calculated using one–way ANOVA with Tukey post–test, * p<0.05, ** p<0.01, *** p<0.001, **** p<0.0001, ns = non–significant.

Moreover, if photo-degradation of 5-MTHF should occur, it does not release 6-FP, nor does it affect MAIT cell activation to any significant extent (39, 54). We hypothesized that the yet uncharacterized pterin photo-metabolite of 5-MTHF may be (*R*/*S*) 5-*N*-methyl-6-FP (5m-6-FP) (**Figure 1D**) (54). Considering the binding mode of 6-FP to MR1 (**Figure 1E**), the 5-*N*-methyl substitution introduces several structural, steric and chiral alterations which may modulate the binding of 5m-6-FP to MR1 and thereby reflect our experimental observations (39). However, *in silico* docking studies predicted that the loss of aromaticity and planarity of the pterin ring, although negatively influencing the π-stacking interaction of both potential 5m-6-FP enantiomers with TYR7, does not impact MR1 binding (cdock affinity scores: -5.9, -6.1 and -6.3 for 6-FP, (*R*)-5m-6-FP and (*S*)-5m-6-FP, respectively; **Figures 2A–C**).

To start resolving whether the inability of 5-MTHF to influence MR1 immunobiology is primarily due to its relative photostability or the fact that its currently unknown pterin photometabolite(s) does not bind to MR1, we exposed both FA and 5-MTHF to UVA *in vitro*, estimated their respective photodegradation by UV-Vis spectrophotometry and quantified the release of inhibitory MR1 ligands by MR1 cell-surface expression and functional MAIT cell inhibition, as previously described (30, 56). As presented in **Figure 1D** and experimentally validated in **Figure 2D**, UVA directly degrades FA (80μM solution in PBS) while the photodegradation of 5-MTHF (80μM solution in PBS) is reactive oxygen species (ROS)-dependent and requires the presence of a photosensitizer (riboflavin; equimolar amounts). When MR1-expressing antigen presenting cells (C1R.MR1) were loaded with UVA-exposed folate (10μM final concentration), only FA led to detectable increases in MR1 cell-surface presentation (**Figure 2E**) and competitive inhibition of 5-OP-RU-mediated activation of a MAIT TCR-expressing cell line (Jurkat.MAIT; **Figure 2F**). Riboflavin, used here as a natural photosensitizer but also reported to be a potential MR1 ligand (57), did not significantly influence MR1 cell-surface expression or Jurkat.MAIT cell activation by itself (**Figures 2E, F**).

Thus, our observations confirm the relative photostability of 5-MTHF, as compared to FA, and suggest that its photodegradation does not release any detectable amounts of antagonistic MR1 ligands. However, more work is required to ascertain the identity and MR1 binding activity of 5-MTHF’s pterin photo-metabolite(s).

## Future directions

Publicly available data are regrettably ill-suited to verify our hypothesis, as they do not consistently distinguish between folate vitamers, or address them individually and therefore only offer equivocal substantiation through cross-study comparison, as in the case of cancer incidence (13, 58). The appropriate testing of our hypothesis requires large double-blind randomized intervention studies directly comparing FA against 5-MTHF supplementation in healthy adults or elderly, as well as in pregnancy, where folate doses of 400-800 μg, up to 5,000 μg for women with increased risk for NTD (59), are being recommended. The primary outcomes should be, according to the target population, any of the observed adverse side effects reported for FA supplementation, such as incidence of allergic disease or cancer. These should be paired with secondary outcomes related to the direct or indirect quantification of MR1-bound 6-FP, such as through mass spectrometry-based proteomic analyses of blood and/or tissues, and *ex vivo* functional assays of isolated MR1-T cells, respectively. Known confounders of folate status such as vitamin B12 deficiency and genetic variations influencing folate metabolism (i.e. methylenetetrahydrofolate reductase (MTHFR) and dihydrofolate reductase (DHFR) polymorphisms) should be included in the recruitment criteria, or at least captured (8). Fitzpatrick skin type and measures of sunlight exposure of study participants require careful recording to assess the UV dose dependence of the primary outcome and folates provided to participants rigorous quality control to ascertain the extent of storage-related degradation, as both factors may have further contributed to the inconsistent findings reported in the literature, according to our hypothesis. Transcriptomic analyses of relevant tissue and/or isolated MR1-T cells, either bulk or at the single-cell level, may be desired to provide evidence of higher resolution and dimensionality. While animal models lend themselves to complementary mechanistic studies, it is important to note that the frequency of MAIT cells is an order of magnitude lower in mice as compared to humans, a problem which could be circumvented using a MAIT cell-rich mouse strain (60), and that the diverse functions of MR1-T cells may not be fully conserved between species.

## Concluding remarks

Previous studies related to folate supplementation have been conducted with the premise that folate-associated health outcomes, whether good or bad, are solely attributable to its function as a vitamin and therefore identical between vitamers (e.g. FA and 5-MTHF). Herein we present the distinct and encouraging hypothesis that current side-effects of folate-fortification are not unavoidable collateral damage but the result of an unfortunate choice of vitamer, and could be simply rectified by using 5-MTHF instead of FA in dietary fortification. If confirmed, our hypothesis will disruptively impact current fortification mandates and the global food and supplement industry, but with far-reaching benefits for human health.

## Materials and methods

### In silico modeling

The MR1 protein (PDB ID:4L4T) was prepared using the Schrodinger protein preparation wizard. Hydrogens were added, hydrogen bonds were optimized and an energy minimization was applied using the OPLS4 forcefield (Protein prep tool: Schrödinger Release 2021-3, Protein Preparation Wizard; Prime, Schrödinger, LLC, New York, NY, 2021). Ligands were manually prepared and docked into the binding site using CovDock (61), using the co-crystallized ligand as the location of binding. To evaluate the model, 6-FP was redocked into the binding site and returned an RMSD of 0.2Å. All visualization were prepared using Maestro: Schrödinger Release 2021-3 (Schrödinger, LLC, New York, NY, 2021).

### Reagents

(6S)-5-Methyl-5,6,7,8-tetrahydrofolate calcium salt hydrate ((6S)-5-MTHF, purity ≥98%) was purchased from Cayman Chemical Limited (Michigan, USA). Folic acid (FA, purity ≥97%), methylglyoxal (MGO, ~40% in H2O, 1.17 g/mL at 25°C), and (-)-riboflavin ((-)-Rb, analytical standard grade) were purchased from Sigma Aldrich (St Louis, Missouri, USA). 6-formylpterin (6-FP, purity ≥95%) was purchased from Schircks Laboratories (Jona, Switzerland). Dulbecco’s phosphate buffered saline (PBS) was purchased from Gibco Life Technologies (NY, USA); CryoPur™ DMSO was from OriGen Biomedical Inc. (Austin, Texas, USA). Stock solutions (1 – 20 mM) of (6S)-5-MTHF, FA and (-)-Rb, 6-FP were prepared in Ar(g)-purged DMSO; aliquoted and immediately frozen at -20°C. In each experiment, an aliquot was thawed and immediately diluted to the required concentration in PBS. All solutions were always kept on ice before use, to avoid degradation of the folate compounds. 5-amino-6-D-ribitylaminouracil (5-A-RU) was produced by synthetic methods based on a published procedure (62). In short, D-ribitylamine and 6-chlorouracil were heated to 170°C in a sealed vessel with triethylamine for 45 min before purification on ion-exchange resin (AG-1X8) gave 6-(D-ribitylamino)uracil. 5-Nitrosylation was achieved by treating 6-(D-ribitylamino)uracil with NaOH and NaNO_2_ at 0°C followed by the addition of aqueous acetic acid. Ion-exchange chromatography (AG-1X8) gave 5-nitroso-6-(D-ribitylamino)uracil which, upon reduction with Na_2_S_2_O_4_, afforded 5-A-RU which was used without further purification.

### UVA exposure and UV-VIS spectrophotometry

Freshly-prepared solutions of 80 µM (6S)-5-MTHF or 80 µM FA were aseptically exposed to UVA radiation on a 24-well plate, (with the lid removed) for exactly 10 minutes at room temperature (approx. 25°C) in the presence or absence of 80 µM (-)-Rb. (-)-Riboflavin was added to the solution of (6S)-5-MTHF or FA, as it can act as a photosensitizer to facilitate and/or enhance the direct photodegradation of either FA (63, 64) or 5-MTHF (65) when exposed to UVA radiation. The UVA source was a handheld 12 LED (wavelength 395 nm) torch. The fluence rates at the sample position were 432 mW/cm^2^. To avoid uncontrolled light exposure, all UVA exposures were performed in dim light as previously described (64, 65).

Following UVA treatment, the concentration(s) of (6S)-5-MTHF and FA were determined by UV-VIS spectrophotometry on a TECAN INFINITE M1000 PRO microplate reader. Absorbance at 295 nm was used to determine the concentrations of (6S)-5-MTHF or FA through interpolation against their respective standard calibration curves (0 – 160 µM).

### Activation studies with MAIT TCR–expressing Jurkat and MR1–overexpressing cell lines

To determine if FA and/or (6S)–5–MTHF photometabolite(s) affect MR1 immunobiology, the effect of each UVA–treated folate solution on MR1 expression and MAIT cell activation was studied *in vitro*, using a co–culture system involving antigen–presenting C1R cells overexpressing MR1 (C1R.MR1 cells) and Jurkat cells expressing a MAIT TCR (“AF7”, originally discovered by Tilloy *et al.* (66)) comprising the TRAV1–2–TRAJ33 α–chain and TRBV6–4 β–chain (Jurkat.MAIT cells), as previously described (30, 56). Both cells were cultured in complete RPMI–1640 (RPMI–1640 medium containing 10% heat–inactivated (56°C, 30 mins) fetal bovine serum and supplemented with Penicillin (100 U/mL) streptomycin (100 μg/mL), L–glutamate (2 nM), 0.5 nM NEAA, 5 nM HEPES, and 50 μM β–mercaptoethanol).

Each UVA–treated (or non–treated) folate solution (in PBS) was diluted 1:4 (v/v) to give 20 µM (6S)–5–MTHF or FA in complete RPMI–1640. In each well of a 96–well U–bottom plate, 30,000 C1R.MR1 cells were co–incubated with 60,000 Jurkat.MAIT cells, in the presence or absence of each folate solution for 1 h at 37°C, 5% CO_2_. Complete RPMI–1640 with 0.2% DMSO and 25% PBS acted as negative control. After the 1 h treatment with each folate solution, Jurkat.MAIT cells were activated by treating the co–cultures for a further 18 h with 5–(2–oxopropylideneamino)–6–D–ribitylaminouracil (5–OP–RU) formed by reacting equimolar amounts (125 nM) of 5–A–RU with MGO in complete RPMI–1640. As controls, some of the wells were treated with 5 or 10 µM 6–FP for 1 h, prior to activation with 5–OP–RU. Subsequently, the cells were processed and stained for flow cytometry. Activation of Jurkat.MAIT cells was measured by an increase in surface CD69 expression. Concurrently, presentation of MR1 ligands was determined by measuring surface translocation of MR1 on C1R.MR1 cells.

### Flow cytometry

Cells were incubated with Zombie NIR™ dye (BioLegend, San Diego, CA, #423106) for 10 minutes in the dark at room temperature, to distinguish live cells from dead cells, and were subsequently washed with FACS buffer. Human Fc–block (BioLegend, San Diego, CA, #422302) was then added to prevent the non–specific binding to FcγR, by incubation for 10 min on ice, in the dark. Cells were then washed in FACS buffer and stained on ice for 20 mins in the dark with a mixture containing the following antibodies prepared in FACS buffer containing 20% Brilliant Stain Buffer (BD, Franklin Lakes, NJ, #563794): anti–CD3 (BD, Franklin Lakes, NJ, #612750), anti–MR1 (BioLegend, San Diego, CA, #361106) and anti–CD69 (BioLegend, San Diego, CA, #3611). Stained cells were then washed twice in FACS buffer, fixed with 2% formaldehyde for 10 mins at room temperature. Finally, the fixed cells were washed once with FACS buffer prior to re–suspension in FACS buffer for flow cytometry analyses on a CyTek™ five laser (355, 405, 488, 561 and 640 nm) Aurora^®^ Flow Cytometer. All flow cytometry data were analysed with FlowJo 10 for Mac (Tree Star, Ashland, OR). Jurkat.MAIT and C1R.MR1 cells were identified as single/live CD3^+^ and single/live CD3^–^GFP^+^ cells, respectively. Expression of CD69 on Jurkat.MAIT cells and MR1 on C1R.MR1 cells were determined as % positive cells and mean fluorescence intensity (MFI), respectively.

## Data availability statement

The original contributions presented in the study are included in the article/supplementary material. Further inquiries can be directed to the corresponding author.

## Author contributions

JT performed all *in vitro* experiments, did the literature search, participated in the discussion of ideas and revised the manuscript. RW did the literature search, participated in the discussion of ideas and revised the manuscript. AC and DO’S participated in the discussion of ideas and revised the manuscript. HA designed the model to predict the binding mode of 6–FP and 5m–6–FP enantiomers to MR1 and participated in the discussion of ideas. OG put forward the hypothesis and wrote the manuscript. All authors contributed to the article and approved the submitted version.

## Funding

This work was supported by the New Zealand Health Research Council Independent Research Organization Fund (AC and OG), the New Zealand Ministry of Business, Innovation and Employment High–Value Nutrition National Science Challenge (JT, DO’S and OG) and the Dines Family Charitable Trust, New Zealand (OG).

## Acknowledgments

We thank Assoc. Prof. Corbett (University of Melbourne, Australia) for kind provision of the Jurkat.MAIT and C1R.MR1 cell lines. The manufacturing of 5–OP–RU was carried out by Prof. Painter’s laboratory (Ferrier Institute, Victoria University of Wellington, New Zealand), financially supported by a program grant from the New Zealand Ministry of Business, Innovation and Employment (award number RTVU1603).

## Conflict of interest

The authors declare that the research was conducted in the absence of any commercial or financial relationships that could be construed as a potential conflict of interest.

## Publisher’s note

All claims expressed in this article are solely those of the authors and do not necessarily represent those of their affiliated organizations, or those of the publisher, the editors and the reviewers. Any product that may be evaluated in this article, or claim that may be made by its manufacturer, is not guaranteed or endorsed by the publisher.
